# The Impact of Training Residents on the Outcome of Robotic-Assisted Sacrocolpopexy

**DOI:** 10.1155/2012/289342

**Published:** 2012-11-01

**Authors:** Mohamed A. Bedaiwy, Mohamed Abdelrahman, Stephanie Deter, Tarek Farghaly, Mahmoud M. Shalaby, Heidi Frasure, Sangeeta Mahajan

**Affiliations:** ^1^Division of Female Pelvic Medicine and Reconstructive Surgery, Departments of Obstetrics and Gynecology and Urology, University Hospitals Case Medical Center, MAC 5034, 11100 Euclid Avenue, Cleveland, OH 44106, USA; ^2^Assiut University, Assiut School of Medicine, Assiut 71111, Egypt

## Abstract

*Objective*. To evaluate the surgical outcomes of robotic-assisted sacrocolpopexy (RASCP) before and after the incorporation of hands-on training for urology and gynecology residents. *Study Design*. Forty-one patients underwent RASCP between December 2008 and March 2010 with one surgeon. RASCP was performed in the context of surgical repair of complex pelvic organ prolapse and/or stress urinary incontinence. The first 20 cases (group I) were performed exclusively by the attending surgeon. In the last 21 cases (group II), the urology resident performed a 50% or more of the RASCP while the gynecology resident performed the supracervical hysterectomy. The primary outcome measure was vaginal vault support at 24 weeks postoperatively based on pelvic organ prolapse quantification (POP-Q). *Results*. Mean ± SD operative time for the entire surgery including RASCP was 282.3 ± 51.3 min and median EBL was 83.1 ± 50.4 mL. Patient demographics and stage of disease did not differ between groups. Procedure time, PACU time, blood loss, and intraoperative complications were similar between groups. Follow-up POP-Q evaluations demonstrated significant correction of all points on vaginal examination for both groups (*P* < 0.001). *Conclusions*. Incorporation of resident training during RASCP allows teaching of robotic surgery techniques in an effective manner without prolonging operative time or affecting the overall surgical outcome.

## 1. Introduction

Pelvic organ prolapse is a very common problem that causes an estimated one in ten women to undergo surgery, and an additional 30% of these women will undergo additional surgery for repeat prolapse [1]. As the population of the United States continues to age, the number of women seeking treatment for pelvic organ prolapse will only continue to grow. The goal of surgical repair of all vaginal vault prolapse is to restore the anatomy and maintain sexual function and durability [2]. While the gold standard for vaginal vault prolapse is an abdominal sacrocolpopexy, large advances have been made in technology to allow minimally invasive approaches to become a viable alternative for surgeons [3]. Additionally, patients are also requesting a minimally invasive approach for their surgery because of the shorter hospital stay, decreased postoperative pain, and better cosmesis [4]. 

Initially, laparoscopy was offered to patients as a mode of performing a minimally invasive sacrocolpopexy. While patients have a decreased morbidity compared to traditional open approaches, there are notable difficulties experienced by the surgeon [3, 5]. Decreased range of motion, two-dimensional vision, and a steep learning curve are some of the many factors that have led to the increased operative time associated with laparoscopic surgery and have limited its widespread adoption by many surgeons. An increased skill level is also required to perform laparoscopic suturing, which is made difficult by the limited range of motion of the laparoscopic instruments [5]. 

More recently, the Da Vinci Surgical System (Intuitive Surgical Inc., Sunnyvale, CA) has provided the features needed to make the minimally invasive sacrocolpopexies successful [6]. The robot offers three-dimensional vision, increased magnification, tremor filtering, and seven degrees of freedom with its instruments that make a robotic-assisted sacrocolpopexy less difficult than using a traditional laparoscope. The technical aspects of a RASCP reflect those of an abdominal sacrocolpopexy [7]. 

As the RASCP becomes more widely adopted into practice, the importance of training the next generation of practitioners becomes apparent without neglecting gaining experience in the traditional abdominal and vaginal hysterectomy concomitant with sacrocolpopexy [8]. Robotic surgery credentials are now required in certain places and in the near future it will be required more widely [9]. The training of residents and fellows on the technique of RASCP is important in both urology [10] and gynecology [11]. Balancing education and patient care is central in any surgery, and careful attention to *primum non nocere* is essential [12]. This study looks to evaluate the outcomes of RASCP before and after the incorporation of hands-on training for urology and gynecology residents.

## 2. Materials and Methods

Data were extracted from the medical records of all patients who underwent robotic-assisted sacrocolpopexy at the University Hospitals Case Medical Center (UHCMC) between April 2008 and March 2010. The approval of the UHCMC Institutional Review Board was obtained. The following data were extracted from each patient's medical record: age; stage of prolapse, concomitant procedure(s), intraoperative and postoperative complications, operative time, blood loss, conversion to laparotomy, length of hospital stay, resident hands- on contribution, and followup. 

Forty-one patients underwent RASCP between December 2008 and March 2010 with one surgeon. RASCP was performed in the context of surgical repair of complex pelvic organ prolapse and, in some patients, stress urinary incontinence. The first 20 cases (group I) were performed exclusively by the attending surgeon. In the last 21 cases (group II), 2 urology residents at the PGY 5 level performed a 50% or more of the RASCP while 2 gynecology residents at the PGY 4 level performed the supracervical or total hysterectomy when indicated.

Prior robotic experience of all surgeons included exposure to didactic and instructional videos encompassing principals of robotic surgeries with video demonstration of a wide variety of gynecologic procedures. Subsequently, a dry laboratory hands-on training with the robotic system was completed. In addition, robotic surgical skills were also acquired in the animal laboratory using the porcine model. 

Concomitantly, all surgeons assisted at the operating table in a wide variety of robotic procedures. Finally, all surgeons participation as console surgeon in the procedures was based on a stepwise progression through various aspects of the surgery by performing tasks with variable complexities under the supervision of the attending surgeons for the 4 residents or the supervision of another experienced attending in a minimum of 15 robotic procedures that were considered as a learning curve. 

## 3. Surgical Technique

After induction of general anaesthesia, patients were positioned in dorsal lithotomy position with both arms tucked by the side and a bean bag was adjusted to keep the arms and the shoulders in place. Pneumoperitoneum is usually induced using a Verres needle. A 12 mm trocar was placed 2–5 cm supraumbilically. Two 8 mm robotic trocars were placed bilaterally, 10 cm lateral to and at the level of the umbilicus. An accessory 10 mm trocar was placed in the left lower quadrant. Monopolar scissors were inserted through the right robotic trocar and a Plasma kinetic (PK) dissecting forceps was inserted through the left robotic trocar.

The peritoneal surface over the sacral promontory was then incised at the base of the sigmoid mesentery and it was carefully dissected down the periosteum to avoid injuring the median sacral vessels. An endoanal sizer was inserted transvaginally to identify the vaginal cuff and the peritoneum overlying the vaginal apex was similarly incised. The bladder was then dissected anteriorly to expose the anterior vaginal wall and the space between the vagina and rectum was dissected in a similar fashion. 

After completing the dissection, a Y-shaped polypropylene mesh (Restorelle, Mypathy Medical, Raynham, MA) was introduced through the 10 mm accessory port. The Monopolar scissors was then changed to a needle driver and the Y-shaped mesh were sutured to the anterior, posterior, and the apex of the vagina using permanent (2–0 Goretex, W. L. Gore and Associates, Inc., Flagstaff, AZ) sutures. The other end of the mesh was then sutured to the sacral promontory using the same type of permanent suture. Afer suturing both ends the mesh was then adjusted to avoid redundancy or excessive tension. CystoUrethoscopic examination after administration of intravenous indigo carmine at the end of the procedure to ensure ureteric patency and bladder integrity was performed in all patients.

## 4. Followup

All patients were asked to come for followup at 6 weeks postoperatively. Subsequent followup visits were individualized thereafter. Records were reviewed up to 24 weeks postoperatively.

## 5. Statistical Analysis

 Patient demographic and clinical characteristics were described among all cases and compared between group 1 cases (without trainee involvement) and group 2 cases (with trainee involvement) by the use of either the chi-square or Fisher's exact test for frequency data or nonparametric Mann-Whitney test. Surgical outcomes were compared between groups in a similar fashion. Preoperative and post-operative POP-Q values were described and comparisons were made between groups by the use of the Mann-Whitney test and analysis of variance. 

## 6. Results

### 6.1. Patient Characteristics

Forty-one patients with stage III/IV prolapse underwent RASCP between December 2008 and March 2010. The first 20 patients were performed exclusively by the attending surgeon (Group I) and the following 21 patients' surgeries were performed by urology or gynecology residents (group 2). Overall, the mean age was 61.5 (15) years and mean BMI was 28.6 (12.7) kg/m^2^. Both groups were comparable regarding their age, ethnicity, and BMI. Stage and history of prior prolapse and incontinence surgery were similar between groups. Eighty-three percent of patients' surgeries were menopausal. Selected comorbidities were present in 12 patients (9 in group 1 and 3 in group 2; *P* = 0.033). Patients' characteristics were summarized in (Table 1).

### 6.2. Intraoperative Outcomes

Concomitant procedures were performed in 36 (88%) patients. When comparing operative outcome measures, there was no significant difference in OR time, procedure time, estimated blood loss, and PACU time between the two groups (Table 2). In addition, bladder perforation was encountered in 1 (2%) of patients of group 1. It was recognized and adequately repaired intraoperatively without adverse sequelae. Vaginal wall was accidentally opened in one patient of group 2 due to extremely thin vagina and was sutured with adequate reapproximation.

### 6.3. Postoperative Outcomes

Postoperative complications are described in Table 2. One patient in group 2 developed postoperative cuff dehiscence and was diagnosed 6 weeks postoperatively during routine postoperative follow-up visit. The vaginal cuff was revisited and adequately sutured under general anesthesia. One patient in group 1 required blood transfusion due to anemia secondary to chronic hemorrhoids in the postoperative period. Two patients in group 1 and one patient in group 2 were readmitted to the hospital for surgical repair of a vaginal mesh extrusion. Mesh extrusion is defined as any vaginal mesh exposure during the follow up period. All erosions were managed by freshening the edges and closing the vaginal defect. One patient required excision of a portion of the exposed mesh. Vaginal estrogen cream was offered to all patients after surgery. Three patients in group 1 developed postoperative urinary tract infection and were properly treated with antibiotics. Prolapse recurrence was reported in one patient of group 1 where the anterior vaginal wall was prolapsed to the level of the hymen. This patient underwent vaginal McCall culdoplasty. One patient in group 2 was complicated by postoperative ileus diagnosed with a CT scan. The patient was managed conservatively and showed a significant improvement on day 6 where she was discharged. One patient in group 2 developed postoperative surgical emphysema and pulmonary edema and she was readmitted to surgical intensive care unit (SICU) where she was properly managed and was discharged after 2 days. The mean length of hospital stay was 1.8 days (range 1–6 days) in both groups. 

Preoperative POP-Q scores were similar between groups for anterior, apex, gh, pb, and TVL values (Table 3). There was a borderline significant difference (*P* = 0.057) between posterior (Ap and Bp) scores between groups. On 12-week followup, the POP-Q values were significantly improved after surgery in both groups (Table 3, time effect) with no effect on vaginal length in both groups (*P* = 0.99). There was no interaction effect between group and time in POP-Q measurements; however, there was limited ability to detect differences due to small sample sizes. 

## 7. Discussion

This study demonstrates that the incorporation of resident training does not appear to affect the immediate operative outcome on performing complex pelvic reconstructive surgery. This is important because the use of robotic-assisted sacrocolpopexy has given patients an alternative treatment to vaginal vault prolapsed [7]. In addition, RASCP is often the only option for patients whose age and medical comorbidities may make them less than ideal candidates for open surgery [7]. Initial studies have shown that initial durability of RASCP is similar to that of abdominal sacrocolpopexies [6]. There is only one study that reported a good patient satisfaction after one year followup after RASCP [13]. More studies are still needed to look at the long-term success of RASCP. 

RASCP is still in its earlier stages of development. There are some negative consequences of RASCP that have emerged including increased mesh extrusion and cuff dehiscence. This is thought to be due to the amount of cautery used at the vaginal cuff particularly if a hysterectomy is done at the time of mesh placement during the RASCP [14]. Approximately 4% of patients will experience dehiscence of the vaginal cuff with the median presentation time of 43 days [2]. Our findings showed only one patient in forty-one (2%) with cuff dehiscence. Advances in the types of mesh and suture used may affect outcomes in the future. 

The limitation of this study is its retrospective design. All data was collected through medical records. This left a potential for misclassification bias, but we would not expect it to be different between the two groups. 

One of the strengths of our study is the use of objective data to determine postoperative outcomes. POP-Q scores determined by the attending physician on 2 occasions (the initial encounter and during the preoperative visit) minimized the bias and discrepancy that could be prevented in the retrospective data. 

As more physicians become trained in RASCP, the technique has been introduced to residents and fellows. While there is agreement that the procedure requires some degree of advanced laparoscopic skills, those used for the robot are often simpler than those used in laparoscopy [3, 6, 15]. The learning curve by the pioneers of RASCP was approximately fifty robotics cases [15]. More recent studies have shown that operative time improves after as few as ten cases [16]. The median operative time reported in our study was 277 minutes. This is similar to other studies that report operative times ranging from 172 minutes to 242 minutes [7]. The increased operative time is not solely related to resident training. The studies with the shortest operative times did not have any concurrent surgeries being performed at the time of the RASCP. This differs largely from our data in which 88% of patients had a concomitant surgery. In agreement with our data, Benson and colleagues reported 284 minutes operative time for Supracervical Robotic-assisted Laparoscopic Sacrocolpopexy versus 194 minutes Robotic-assisted Laparoscopic Sacrocolpopexy [17]. In the future, it might be possible to compare patients undergoing only RASCP to obtain a more accurate time of resident operative times. 

Minimally invasive surgery will only become more common in the future [1]. Residency training programs must use all opportunities to train residents and fellows on robotic surgery [16]. The quicker learning curve of the robot allows residents and fellows the chance to adopt the techniques they learn while in training and apply them in their future practices. As pelvic organ prolapse surgery volume increases, RASCP provides residents and fellows with an excellent opportunity to train on the robot safely and feasibly in a manner that does not affect patient morbidity [8, 12]. Long-term data and robotic training consoles will only help in the development of such clinical training.

## Figures and Tables

**Table 1:** Patient/clinical demographics overall and by group, *P* value is comparison between groups.

	Overall	Group 1 (*n* = 20)	Group 2 (*n* = 21)	*P* value
Age (years), median (IQR)	61.5 (15)	61 (12)	63 (16)	.744
Race				.395
Caucasian	29 (71%)	12 (60%)	17 (81%)	
African American	4 (10%)	3 (15%)	1 (5%)	
Hispanic	1 (2%)	1 (5%)	0	
Unknown	7 (17%)	4 (20%)	3 (14%)	
BMI, mean (SD)	28.6 (12.7)	29.0 (25.9)	27.1 (9.3)	.754
Stage				.488
III	39 (95%)	20 (100%)	19 (91%)	
IV	2 (5%)	0	2 (9%)	
Prior prolapse surgery (yes)	2 (5%)	0	2 (10%)	.488
Prior incontinence surgery (yes)	6 (15%)	2 (10%)	4 (19%)	.663
Burch	1	1	0	
TVT	2	0	2	
Rectus FS	1	1	0	
Other	2	0	2	
Menopause (yes)	34 (83%)	18 (90%)	16 (76%)	.410
Selected comorbidities				
Diabetes	3 (7%)	2 (10%)	1 (5%)	
COPD/Asthma	2 (5%)	2 (10%)	0	
CAD	1 (2%)	1 (5%)	0	
HTN	10 (24%)	8 (40%)	2 (10%)	
Cancer	4 (10%)	4 (20%)	0	
Current steroid use	1 (2%)	1 (5%)	0	.488

**Table 2:** Surgical outcomes overall and by group.

	Overall (*n* = 41)	Group 1 (*n* = 20)	Group 2 (*n* = 21)	*P* value
Concomitant procedures	36 (88%)	18 (90%)	18 (86%)	.999
TVT	27 (66%)	12 (60%)	15 (71%)	
SCH	22 (54%)	12 (60%)	10 (48%)	
Posterior repair	9 (22%)	2 (10%)	7 (33%)	
Perineorraphy	8 (19%)	0	8 (38%)	
TAH	5 (12%)	3 (15%)	2 (10%)	
Enterocele	1 (2%)	1 (5%)	0	
Other	1 (2%)	1 (5%)	1 (5%)	
OR time (min), median (IQR)	328.5 (56)	320 (38)	336 (85)	.283
Range	241–506			
Procedure time (min), median (IQR)	277 (65)	257 (53)	283 (86)	.708
Range	182–426			
PACU time (min), median (IQR)	97.5 (61)	90 (80)	110 (45)	.444
Range	52–335			
Uterine weight (g), mean (SD), *n* = 19	72.7 (66.9)			
EBL (cc), median (IQR)	50 (50)	75 (50)	50 (75)	.922
Range	5–200	25–175	5–200	
Hgb, *n* = 22				
Pre-operative	13.2			
Post-operative	11.4			
HCT, *n* = 22				
Pre-operative	40.8			
Post-operative	34.7			
Intraoperative complications				.948
Perforation (bladder)	1	1	0	
Vaginal wall defect	1	0	1	
Post-operative complications				
Urinary retention	15	7	8	
Fever	2	0	2	
Readmission to hospital	3	2	1	
UTI	3	3	0	
Transfusion	1	1	0	
Cuff dehiscence	1	0	1	
Failed voiding	1	1	0	
Ileus	1	0	1	
Prolapse recurrence	1	1	0	
Emphysema, pulmonary edema	1	0	1	

**Table 3:** Mean preoperative and postoperative POP-Q values by group.

	Preoperative^*≠*^		Postoperative		*P* value
	Group 1	Group 2	Group 1	Group 2	(time effect)
Anterior					
Aa	+2.3	+1.6	−2.7	−2.9	<.001
Ba	+3.6	+2.7	−2.7	−2.9	<.001
Apex					
C	−2.6	−2.1	−9.7	−9.8	<.001
Posterior					
Ap	−1.7	−0.5	−2.5	−2.7	<.001
Bp	−1.7	−0.2	−2.5	−2.8	<.001
gh	+3.8	+3.7	+3.2	+3.1	<.001
pb	+2.4	+2.2	+3.3	+3.3	<.001
TVL	+9.6	+9.6	+9.6	+9.8	0.99

^*≠*^ Pre-operative POP-Q scores were similar between groups. However, the closest to a borderline significant difference was in the posterior wall (Ap and Bp) scores (*P* = .057).
